# MUC16: clinical targets with great potential

**DOI:** 10.1007/s10238-024-01365-5

**Published:** 2024-05-17

**Authors:** Xin-Yu Zhang, Lian-Lian Hong, Zhi-qiang Ling

**Affiliations:** 1 Zhejiang Cancer Institute, Zhejiang Cancer Hospital, No.1 Banshan East Rd., Gongshu District, Hangzhou, 310022 Zhejiang China; 2 Hangzhou Institute of Medicine (HIM), Chinese Academy of Sciences, Hangzhou, 310018 Zhejiang China; 3 The Second Clinical Medical College of Zhejiang, Chinese Medicine University, Hangzhou, 310053 China

**Keywords:** Mucin 16 (MUC16), CA125, Immune escape, Clinical trials, Biomarker

## Abstract

Mucin 16 (MUC16) is a membrane-bound mucin that is abnormally expressed or mutated in a variety of diseases, especially tumors, while being expressed in normal body epithelium. MUC16 and its extracellular components are often important cancer-related biomarkers. Abnormal expression of MUC16 promotes tumor progression through mesenchymal protein, PI3K/AKT pathway, JAK2/STAT3 pathway, ERK/FBW7/c-Myc, and other mechanisms, and plays an important role in the occurrence and development of tumors. In addition, MUC16 also helps tumor immune escape by inhibiting T cells and NK cells. Many drugs and trials targeting MUC16 have been developed, and MUC16 may be a new direction for future treatments. In this paper, the mechanism of action of MUC16 in the development of cancer, especially in the immune escape of tumor, is introduced in detail, indicating the potential of MUC16 in clinical treatment.

## Introduction

The MUC16 gene and its homologous antigen carbohydrate antigen 125 (CA125) have long served as classic markers for ovarian cancer [1, 2]. However, CA125 is not exclusive to ovarian cancer, being expressed in normal tissues and benign diseases [3–5] with differing mechanisms. The low specificity of CA125 poses a challenge in clinical diagnostics, urging the need for enhanced accuracy. Comprehensive understanding of MUC16's role in disease progression is crucial for precise treatment. This detailed exploration elucidates the intricate mechanisms of MUC16/CA125, highlighting the potential of CA125 as a clinical marker and underscoring MUC16/CA125's substantial promise as a diagnostic tool and therapeutic target.

## Structure of MUC16

**Fig. 1:**
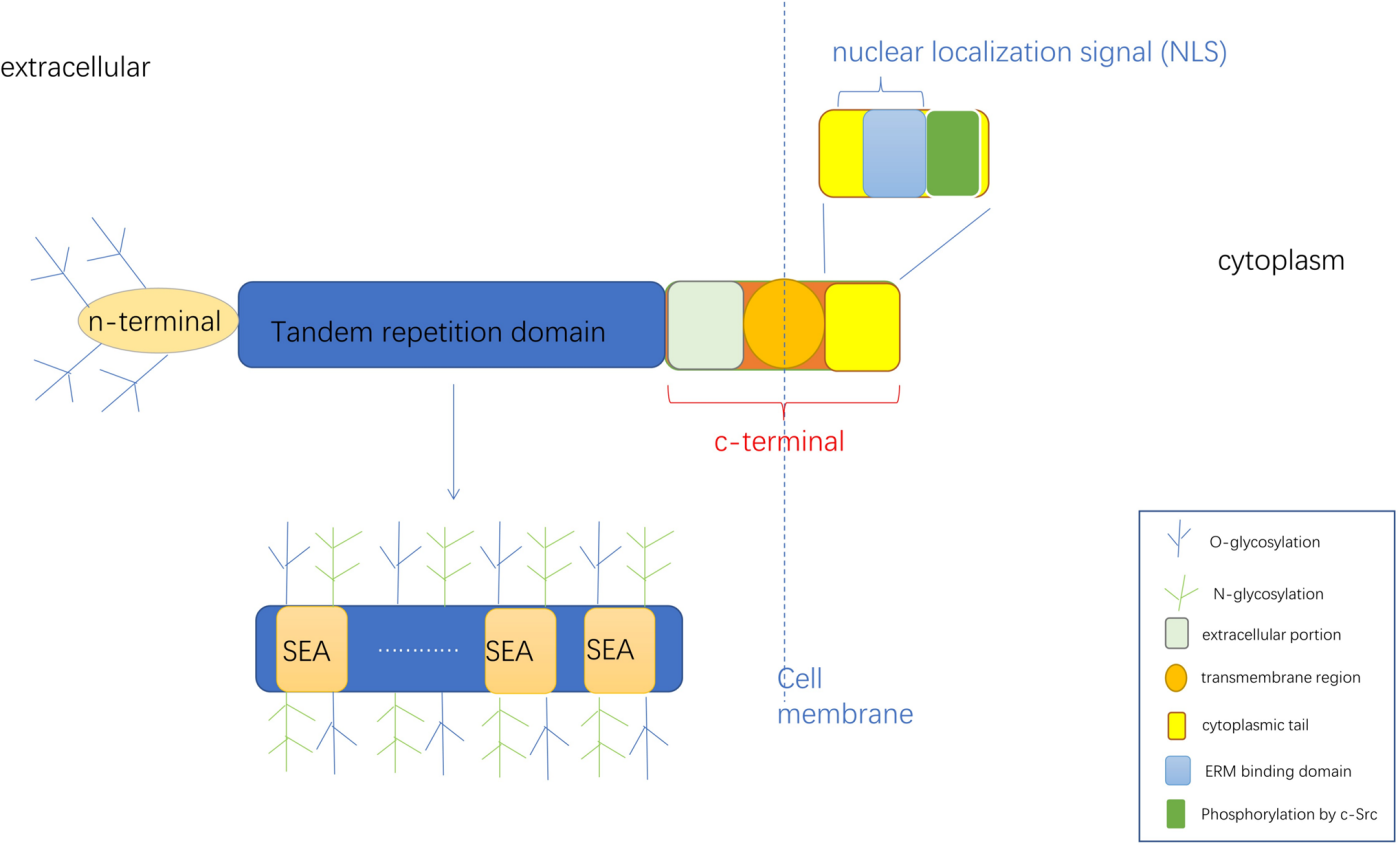
Structure diagram of MUC16. MUC16 features three domains: N-terminal, carboxyl-terminal, and tandem repeating domain. The latter contains 16 SEA modules. The carboxyl-terminal domain includes an extracellular part, a transmembrane region, and a short cytoplasmic tail with a potential NLS, an ERM binding domain and a phosphorylated tyrosine. Both *O*-linked and *N*-linked glycosylation occurs, with the amino-terminal domain dominated by O-glycosylation

MUC16 is a type I transmembrane mucin, a membrane-bound mucin, and the largest mucin ever discovered [6]. MUC16 was first recognized as CA125, which is a classic marker for ovarian cancer. Its gene is present on the short arm of human chromosome 19p13.2, which is 179 kb long. MUC16 consists of 22,152 amino acids with a core protein size of about 2–5 × 10^6^ Da [7], and the predicted molecular mass of glycosylated mucins is estimated to exceed 5 MDa [8]. MUC16 consists of three main domains: an extracellular serine/threonine-rich amino terminal domain, a carboxyl terminal domain, and a tandem repeating domain which is a major part of muc16's molecular structure [6, 9] (Fig. 1).

The sea urchin sperm, enterokinase, and agrin (SEA) domains in the series duplication region are common modules in sperm proteins, enterokinase, and agglomerin of sea urchins. The three-dimensional structure of the SEA domain is an α/β sandwich fold, which is divided into a layer consisting of four antiparallel β slices and a short α helix, and a second layer consisting of two long α helices and two short β slices. There are 16 SEA modules in MUC16 [6], and the SEA region is composed of multiple repeats. The putative epitope of anti-MUC16 antibodies is located in the polypeptide portion of MUC16 [10].

The carboxy-terminal domain consists of the extracellular portion, the transmembrane region, and the short cytoplasmic tail which contains 32 amino acid residues including a possible nuclear localization signal (NLS), an Ezrin/radixin/moesin (ERM) binding domain, and a tyrosine residue that has been phosphorylated by c-Src [2, 9].

The amino terminal domain consists of 12,068 amino groups [7], rich in serine, threonine, and proline, and accounts for the majority of known glycosylation in MUC16 [9]. The core extracellular domain of MUC16 protein has a high ability of *O*-glycosylation. There are mainly two types of glycosylation in MUC16:*O*-linked and *N*-linked, in which the n-terminal domain is mainly determined by the ability of *O*-glycosylation [9, 11]. *N*-linked oligosaccharides on MUC16 can be divided into two main categories: para-compound oligosaccharides and high-mannose oligosaccharides. High mannose-type oligosaccharides can be recognized by the cell surface lectin DC-SIGN (dendritic cell-specific ICAM-grabbing non-integrin), which may be one of the mechanisms of MUC16 evasive immune response, although this conclusion has not been proven so far [12].

Both amino terminal domains and tandem repeat domains are anchored by a relatively short carboxyl terminal containing a transmembrane region and a short cytoplasmic tail [7]. Muc16 is anchored to the surface of tumor cells or epithelial cells by transmembrane regions and a short cytoplasmic tail [9]. When part of MUC16 is sheared and released into the blood, it becomes CA125.

## MUC16 as a biomarker of disease

MUC16 was first detected in ovarian cancer as CA125, which is considered as a classic biomarker for ovarian cancer [1]. MUC16 has been expressed in a variety of cancers, such as pancreatic cancer, and can be used as a marker for pan-cancer. Meanwhile, it can also be used as an early identification marker in some diseases. For example, muc16 (CA-125) level is considered to be a marker associated with idiopathic pulmonary fibrosis (IPF), which is the most common interstitial lung disease (ILD) [13]. It was also reported that CA-125 was increased in rheumatoid arthritis-related interstitial lung disease (RA-ILD), and CA125 level was closely related to the incidence and severity of ILD in RA patients [5], and CA125 level could also be used as a marker for possible cancer in patients with ILD (Table 1) [14].

**Table 1:** MUC16/CA125 as a clinical marker

Disease species	Expression situation	Representative meaning
Interstitial lung disease	CA125 expression was upregulated	Disease marker
Ovarian cancer	Overexpression	Cancer marker Represents a poor prognosis
Ovarian cancer	CA125 expression was upregulated 3–6 months after treatment	The patient did not respond well to primary treatment
Endometrial carcinoma	HE4 and CA125 increased	Predicting high LNM risk in patients with endometrioid adenocarcinoma before surgery
Epithelial ovarian cancer	CA125 and HE4 were cleared slowly after chemotherapy	Suggests that the patient may be resistant to platinum drugs and the prognosis is poor
Pancreatic cancer	Overexpression	Cancer marker
Acute heart failure	CA125 expression was up-regulated	Represents a poor prognosis
Cancer of unknown primary	Mutation	Cancer marker
EBV-associated lymphoepithelioma-like cholangiocarcinoma	Mutation	Help identify this particular subtype
Primary malignant melanoma of the esophagus	Mutation	Help identify this particular subtype
Pseudomyxoma peritonei	Mutation	Help identify this particular subtype
Ovarian serous cystadenocarcinoma	The RNA levels of MUC16, PAX8 and SOX17 genes were changed	Distinguish ovarian serous cystadenocarcinoma from other tumor types
Gastric cancer	Mutation	Represents higher TML and longer median survival, and higher response to immunotherapy
Colon cancer	Mutation	Represents high TMB
Non-small cell lung cancer	Mutation	It is associated with increased mutation load TMB and neoantigen load It indicates better effect of ICI treatment
Melanoma	Mutation	Represents high TMB It indicates better effect of ICI treatment

In recent years, MUC16 mutations have gradually been found to be a marker for the early identification of cancer. For example, MUC16 is one of the most common mutations in patients with cancer of unknown primary (CUP) [15] and leiomyosarcoma [16] treated with eribulin and dacarbazine. MUC16 mutations are often detected in Epstein–Barr virus (EBV) associated lymphoepithelioma-like cholangiocarcinoma (EBV-LELCC), which is a rare subtype of intrahepatic cholangiocarcinoma (IHCC) [17] and in the rare disease primary malignant melanoma of the esophagus (PMME) [18], suggesting that the patient may be a special type of cancer. There is also a high frequency of MUC16 mutations in Pseudomyxoma peritonei (PMP) of ovarian origin [19]. MUC16, PAX8, and SOX17, whose RNA quantity can distinguish ovarian serous cystadenocarcinoma (OSCA) from other tumor types [20].

CA125 level alone is an important criterion for early tumor screening, and combined with other criteria can improve the efficiency of tumor detection. Studies have shown that the GAL3ST2 rs12469459 mutation can affect the level of CA125, which improves the diagnostic performance of CA125 for ovarian cancer. Similarly, CA125 levels associated with single-nucleotide polymorphisms (SNPs) may improve the accuracy of CA125 in diagnosing PDAC patients [21]. Because higher antibody levels may mask the detection of circulating antigen, the combination of circulating CA125 levels with anti-CA125 antibodies has been shown to be more effective than CA125 alone in detecting early epithelial ovarian cancer (EOC) [22].

Muc16 predicts a poor prognosis in EOC [23]. Discontinuous patterns of congestive intrarenal venous flow (IRVF) (biphasic and uniphasic) predict adverse outcomes in acute heart failure (AHF), which is difficult to detect in patients with severe symptoms. The upregulation of CA125 showed a nonlinear positive correlation with IRVF mode of AHF, which could represent IRVF mode as a marker of AHF deterioration [24]. CA125 was a more accurate predictor of long-term mortality than N-terminal pro-Brain natriuretic peptide (NT-proBNP), which is the marker for heart failure (HF) in patients with both AHF and severe functional tricususal regurgitation (TR) and can be used as a biomarker for risk stratification [25]. CA125 can also be used as an independent predictor of all-cause mortality in HF after correction for BIOSTAT risk model (age, blood urea nitrogen (BUN), NT-proBNP, hemoglobin, and beta-blocker) [26]. Mucin variants and its splice variants (SV) are an important marker for the prognosis of ductal carcinoma of the pancreas (PDAC). The high expression of MUC4 variants and full-length transcripts of MUC16 suggested a shorter survival in the variants [27]. An increase of CA-125 concentration > 5U/mL within the normal range at 3 and 6 months after treatment in ovarian cancer patients represents an adverse prognostic factor for a complete response to primary therapy [28].

MUC16 was also effective in risk stratification of patients and evaluation of therapeutic efficacy. In patients with endometrial cancer (EC), human epididymis 4 (HE4) and CA125 were positively correlated with high risk factors such as lymphatic space infiltration (LVSI), grade, and lymph node metastasis (LNM). The combination of HE4 and CA125 can predict the high LNM risk of patients with endometrioid adenocarcinoma before surgery, which facilitates preoperative risk stratification [29]. In patients with EOC, the combination of CA125 and HE4 can identify most platinum-sensitive individuals. If CA125 and HE4 clearance is slow after chemotherapy, it indicates that patients may be resistant to platinum-based drugs and have a poor prognosis [30].

MUC16 has recently been found to have a strong association with tumor mutation burden (TMB). Xianyu Hu et al. identified two molecular subtypes CS1 and CS2 of gastric cancer by multiomics method, among which the CS2 group with high TMB, more mutations, more CNAs and higher response to immunotherapy had higher MUC16 mutation and higher MUC16 mutation predicted better prognosis [31]. MUC16 mutation may be an independent influencing factor for tumors with higher tumor mutation load (TML) and longer median survival, and may be a new means to predict the sensitivity of anti-programmed death 1 (PD-1) therapy [32]. MUC16 mutations also represent high TMB in melanoma [33]. The mutation status and number of MUC4, MUC16, and Titin (TTN) are also a combination that can better predict TMB and Overall survival (OS) [34]. TTN and MUC16 also showed a higher mutation frequency in colon cancer patients with high tumor immune microenvironment (TIM), which also represented a higher TMB [35].

In the realms of non-small cell lung cancer (NSCLC) and melanoma, the MUC16 gene mutation demonstrates a significant correlation with tumor mutation burden (TMB) and neoantigen load. Tumors with MUC16 mutations exhibit heightened immunogenicity, reflected by an increased proportion of CD8A and Programmed cell death 1 ligand 1 (PD-L1)-positive cells in the tumor microenvironment compared to wild-type tumors. Additionally, the expression of multiple inhibitory immune checkpoint markers is elevated in MUC16-mutant tumors. Furthermore, MUC16-mutant tumors show an enrichment of various immune response genes, underscoring their importance in eliciting robust immune reactions.

In non-small cell lung cancer (NSCLC) and melanoma cohorts, patients with MUC16 variants display superior outcomes with immune checkpoint inhibitor (ICI) therapy compared to wild-type patients. This suggests that MUC16 mutations may serve as potential biomarkers guiding ICI treatment. Moreover, a reduction in CA125 levels is indicative of a favorable prognosis in late-stage NSCLC patients undergoing ICI therapy [36, 37]. This provides crucial research leads for future personalized cancer therapy and immunotherapy directions. However, further in-depth research and validation are essential to confirm whether MUC16 mutations reliably serve as effective markers for immunotherapy [38].

## Expression of MUC16 in normal tissues

MUC16, a protein widely expressed in various organ epithelia, serves essential functions in maintaining mucosal integrity and providing lubrication [39]. It plays a crucial role in forming microfolds in corneal and conjunctival cells, achieved by binding to the N-terminus of the ERM protein family via its cytoplasmic tail polyamino acid sequence (RRRKK). Additionally, MUC16 acts as a protective barrier against pathogens, such as Staphylococcus aureus, safeguarding human limbal epithelium (HCLE) cells [40]. Furthermore, MUC16 is expressed on the apical surface of endometrial epithelial cells, playing a protective role. Its significant increase during the menstrual cycle corresponds to the rise in endometrial epithelial content during the secretory phase [41].

In glycosylation processes, MUC16 enhances binding to the G0 glycotype of the Fc portion of immunoglobulin, contributing to the accumulation of specific antigens within the MUC16-expressing upper calyx. This strengthens the glycocalyx barrier function by adding a layer of pathogen-specific antibodies, effectively trapping pathogens like human immunodeficiency virus (HIV) and neutralizing them through immune rejection mechanisms. Researchers may explore optimizing vaccine effectiveness by enhancing MUC16 binding for this purpose [42].

## Function and mechanism of MUC16 in disease

MUC16 is not only expressed in normal tissues, but also plays a role in various diseases, especially ovarian cancer and pancreatic cancer. MUC16 is often overexpressed in tumors, and highly expressed MUC16 promotes tumor progression through various mechanisms.

### Ovarian cancer

Expression of MUC16 is elevated in epithelial ovarian cancer [23].

**Fig. 2:**
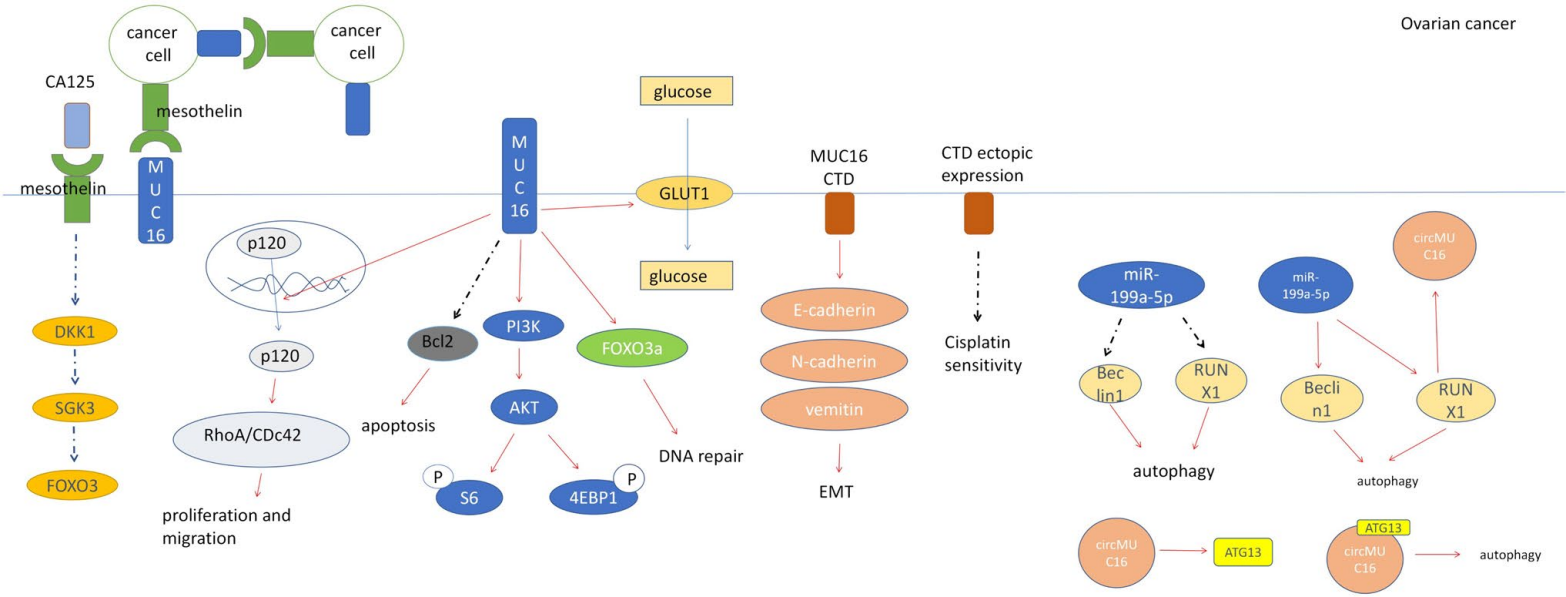
Mechanism of action of MUC16 in ovarian cancer. Free CA125 binding MSLN inhibited DKK1 expression, activated SGK3/FOXO3a pathway, and MUC16 binding MSLN on cell surface promoted epithelial ovarian cancer metastasis. High expression of MUC16 can promote the development of ovarian cancer through Foxo3a, Bcl-2, GLUT1, E-cadherin, P120ctn/RhoA, and PI3K/AKT/pS6.CircMUC16 modulates autophagy by sponging miR-199a-5p to enhance Beclin1 and RUNX1 expression, reciprocally regulated by RUNX1, and directly interacts with ATG13 to influence ATG13 expression

Both MUC16 and mesothelin (MSLN) are highly expressed in epithelial ovarian tumors, and both soluble and cell surface-associated forms of native MUC16 interact with MSLN. Binding between tumor cells via MSLN-MUC16 interaction—the *n*-linked glycochain of MUC16 is a necessary condition for interaction—may provide the basis for epithelial ovarian cancer metastasis [43]. Binding of free CA125 to MSLN inhibits Dickkopf-related protein 1 (DKK1) expression to enhance migration and activate the  serum and glucocorticoid-regulated kinase 3 (SGK3)/forkhead box O3 (FOXO3a) pathway (Fig. 2) [44].

MUC16 enhances glucose uptake by tumor cells by promoting the expression of glucose transporter 1 (GLUT1), a glucose transporter that promotes the transport of glucose molecules across cell membranes, and promotes glycogen synthesis, thus enabling tumor cells to produce more energy for proliferation [23]. The effect of MUC16 on the inhibition of anti-apoptotic B-cell lymphoma 2 (Bcl-2) suggests that MUC16 controls AKT Serine/Threonine Kinase 1 (AKT) regulated apoptotic signaling. Phosphorylation of S6 and 4EBP1 as downstream effector molecules involved in translational regulation of the phosphatidylinositol 3'-kinase (PI3K)/AKT pathway was reduced in ovarian cancer cell lines with MUC16 knockout, but not in breast cancer. Based on these results, we suggest that MUC16 regulates tumor growth at least in part by activating the PI3K/AKT pathway [45].

High expression of MUC16 promotes the translocation of p120-catenin (P120ctn) to the cytoplasm but does not affect the total protein expression level of p120ctn which may act through the cytoplasmic tail (CTD) of MUC16 increasing the expression level of cytoplasm p120ctn and activates ras homolog (Rho) GTPases RhoA/Cdc42 to promote proliferation and migration of EOC cells [46]. The expression of MUC16CTD can promote the decrease of E-cadherin expression, the increase of *n*-cadherin and vimentin expression, and promote the distant metastasis of ovarian cancer cells. The expression of CTD enhances the growth, movement, invasion, and metastasis of ovarian cancer cells. In vitro experiments have also demonstrated that MUC16CTD can enhance tumorigenicity [47].

Circular MUC16 (CircMUC16) is overexpressed in epithelial ovarian cancer tissues, and its expression is related to the stage and grade of ovarian cancer. CircMUC16 is overexpressed in epithelial ovarian cancer. CircMUC16 regulates Beclin1 and RUNX Family Transcription Factor 1 (RUNX1) through sponging miR-199a-5p. Circmuc16 affects autophagy by binding directly to △475–526 region of Autophagy Related 13 (ATG13) protein to promoting the expression of ATG13. Meanwhile, RUNX1 can upregulates CircMUC16 expression by promoting its transcription. CircMUC16-mediated autophagy exacerbates EOC invasion and metastasis. These results suggest that CircMUC16 may be a potential target for the diagnosis and treatment of epithelial ovarian cancer [48].

In NIH: OVCAR3 cells, downregulation of MUC16 on the cell surface reduces DNA repair, increases DNA damage caused by genotoxic drugs, and selectively sensitizes tumor cells to genotoxic drugs by downregulating FOXO3a expression levels and reducing FOXO3a nuclear localization. Ectopic expression of MUC16CTD, on the other hand, decreased cisplatin sensitivity and caspase activity neither had any effect on sensitivity to taxol [49].

### Pancreatic cancer

MUC16 is overexpressed in pancreatic cancer, although hardly expressed in normal pancreatic ducts. MUC16 can promote the proliferation, invasion and migration of pancreatic ductal carcinoma in a variety of ways.

**Fig. 3:**
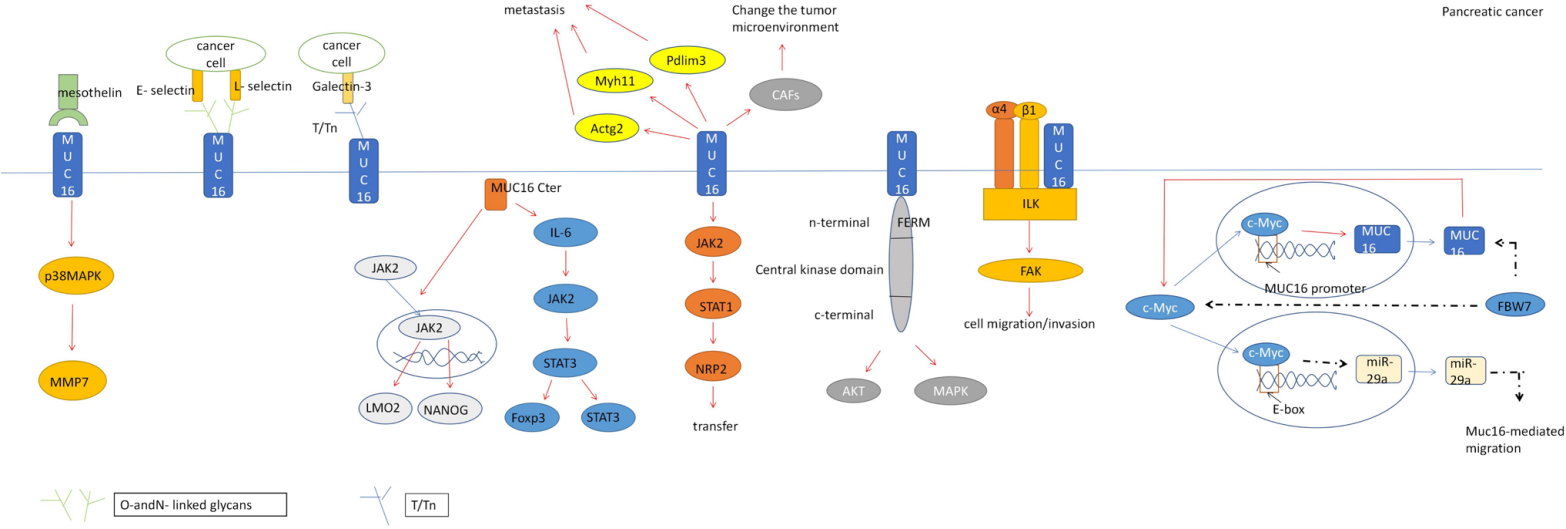
Mechanism of action of MUC16 in pancreatic cancer. Interacting with Mesothelin, MUC16 enhances MMP7 expression via p38MAPK phosphorylation. Sialosylated MUC16 binds E- and L-selectin, promoting adhesion to host tissues. galectin binding facilitates PDAC cell metastasis.MUC16-Cter induces JAK2 nuclear shift, upregulating LMO2 and NANOG. Cytoplasmic MUC16c release activates IL-6 secretion through JAK2/STAT3.Cytoskeletal proteins (Actg2, MYH11, Pdlim3) are upregulated, altering the tumor microenvironment. NRP2 upregulation via JAK2/STAT1 promotes metastasis.MUC16 binds FAK, activating downstream AKT and ERK/MAPK. Truncated *O*-glycochains interact with integrins, activating ILK.c-Myc regulates MUC16 expression transcriptionally, and forming a feedback loop.MiR-29a antagonizes MUC16-mediated migration and invasion

MUC16 was co-expressed and interacted with mesothelin proteins in both ovarian epithelial tumors [43] and pancreatic ductal carcinoma [50], and the combination of the two promoted cancer metastasis and invasion. In pancreatic cancer, MUC16 can be combined with Mesothelin to increase the expression of matrix metalloproteinase-7 (MMP7) by increasing the phosphorylation of p38 mitogen-activated protein kinase (MAPK) (but not changing the total p38 level) to promote the movement and invasion of pancreatic cancer cells (Fig. 3) [50].

Loss of MUC16 reduces the occurrence and metastasis of KRAS-induced pancreatic ductal adenocarcinoma by altering tumor microenvironmental factors. Deletion of MUC16 in mice with the activating mutations Kras^G12D/ +^ and Trp53^R172H/ +^ significantly slowed disease progression and extended overall survival time. MUC16 promotes epithelial mesenchymal transformation (EMT) and PDAC cell metastasis by upregulating the expression of cytoskeletal proteins Actg2, MYH11, and Pdlim3, and has been found to alter the tumor microenvironment in pancreatic cancer progression [51].

In highly metastatic pancreatic cancer, mutated Kras^G12D^ induces upregulation of MUC16 by ERK/F-box and WD repeat dominium containing 7 (FBW7)/c-Myc axis. The Kras mutation activates ERK, resulting in the instability of FBW7 and the up-regulation of c-Myc [52]. c-Myc can bind to the promoter of MUC16 and activate muc16 expression transcriptionally, directly or indirectly promote the up-regulation of MUC16 expression and CA125 shed, further mediating the metastasis of pancreatic cancer cells. Meanwhile, MUC16 can also activate c-Myc in pancreatic cancer, up-regulate the expression of c-Myc, enhance the binding of c-Myc to E-box in the promoter of microRNA-29a (miR-29a) (which is the downstream target of FBW7 gene), and inhibit the transcription of miR-29a. miR-29a could antagonize MUC16-mediated migration and invasion. MUC16/c-Myc forms a positive feedback loop in highly metastatic pancreatic cancer to maintain high serum CA125 levels [53].

As pancreatic cancer progresses, The *O*-glycochain structure of some branches is truncated to produce tumor-specific short mycin types *O*-glycochain TN (GalNAc α1-O-Ser/Thr) and sialyl Tn (Sialic acid α2-6 GalNAc α1-O-Ser/Thr), structural expression of truncated *O*-glycochains (TN and STN antigens) on mucins enhances the malignant potential of PDAC cells. muc16 is an abnormal o-glycosylated protein detected in tumors expressing truncated *O*-glycochains. In PDAC, the deletion of glycosyltransferase Core 1 Synthase, Glycoprotein-N-Acetylgalactosamine 3-Beta-Galactosyltransferase 1 (C1GALT1) and its molecular chaperone Cosmc are both mechanisms leading to increased expression of truncated *O*-glycochains and increased tumor aggressiveness. In PDAC cells, the deletion of C1GALT1 gene resulted in a significant increase of TN in MUC16 mucin. The increased expression of truncated *O*-polysaccharide (TN-polysaccharide) on muc16 increases the proliferation rate of tumor cells and is faster than that of stromal cells, which reduces the fibrosis of PDAC tumors, that is, they have fewer stromal cells and are more aggressive, which promotes the progression and metastasis of tumors [54]. MUC16 can interact with ErbB (epidermal growth factor) receptors to promote the progression of PDAC by activating AKT/Glycogen Synthase Kinase 3 Beta (GSK3b) oncogenic signaling, which is enhanced by abnormal glycoylation, and AKT/GSK3b oncogenic signaling further enhances the abnormal glycotype of MUC16, forming a positive feedback. These evidences to some extent support the hypothesis that EGF-like domains on MUC16 bind and activate ErbB receptors by glycation regulation, though this hypothesis has not been fully tested [55].

Muc16 can interact with β-galactoside binding lectins to promote PDAC cell metastasis [56, 57], such as Galectin-1 and Galectin-3 proteins, which can reduce the adhesion ability of MUC16 gene knockout cells. The expression of tumor-associated antigens Thomsen–Friedenreich (TF/T) and Thomsen–nouvelle (Tn) which is the first selectors of galectin receptors in PDAC cells decreased after MUC16 knockout, and their reduced binding to galectins may be attributed to reduced expression of MUC16-associated polysaccharides. In addition to Mesothelin and Galectin-3, MUC16 also interacts with focal adhesion kinase (FAK). MUC16 binds to the four-point-one, ezrin, radixin, moesin (FERM) domain of FAK and activates FAK at the local adhesion, further promoting downstream activation of FaK-mediated Akt and ERK/MAPK. The interaction of MUC16 with FAK may play an important role in cytoskeletal protein rearrangement, thus promoting cell metastasis. It has been found that MUC16 containing truncated *O*-glycochains enhances migration of PDAC cells by specifically interacting with α4 and β1 integrin complexes on cancer cell membranes to activate the integrin-Fak signaling axis of integrin-linked kinase (ILK), which is enhanced by abnormal glycation structures [58]. Upregulation of sialosylated MUC16 may also specifically bind E- and L-selectin, which is related to the sialosylated structure of muc16 on the *O*- and *N*-linked sugar chains and enhance the binding ability of E- and L-selectin ligands to host tissues, promoting the metastasis of PDAC cells [59].

In PC cells, the carboxyl terminal of MUC16 can be cleaved to produce a 17 kDa cleaved MUC16 (MUC16-cter), Janus Kinase 2 (JAK2) nuclear shift induced by MUC16-Cter can upregulate stem specific genes such as LIM domain Only 2 (LMO2) and Nanog Homeobox (NANOG) to promote proliferation, metastasis and chemotherapeutic resistance of PC cells (reduced cytotoxic response to chemotherapy drugs) [60]. At the same time, induced release of MUC16c into the cytoplasm also promotes the secretion of IL-6, which activates the JAK2/STAT3 pathway, promotes the expression of Foxp3 in tumor tissues and the abundance of tumor-associated regulatory T cells (Tregs) [61]. Unlike MUC16 knockdown reduction, which significantly reduced colony formation and migration, ectopic overexpression of MUC16-Cter showed a significant increase in colony formation and movement in MiaPaCa2 pancreatic cancer cells. MUC16 upregulates Neuropilin 2 (NRP2) through JAK2 / STAT1 signaling in PDAC and promotes metastasis of pancreatic cancer [62].

### Lung disease

**Fig. 4:**
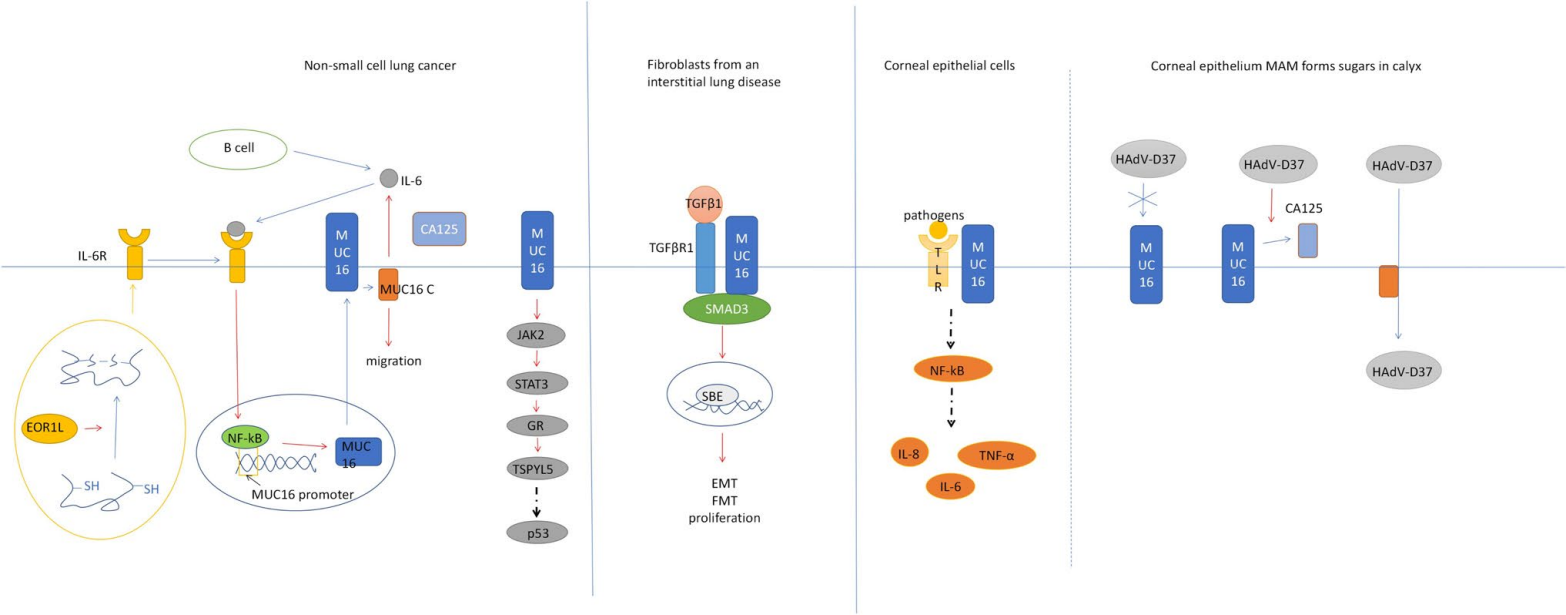
Mechanism of action of MUC16 in non-small cell lung cancer, interstitial lung disease and corneal epithelial infection. In NSCLC, ERO1L influences IL6R secretion, activating NF-κB, and promoting MUC16 expression. MUC16, in turn, regulates TSPYL5 via JAK2/STAT3/GR, impacting p53 expression. In interstitial lung disease, TGF-β1-induced MUC16 complexes with pSmad3 drive fibroblast proliferation and cellular transformation. Conversely, in corneal epithelial cells, MUC16 inhibits pro-inflammatory cytokines via TLR2/TLR5. HAdV-D37 compromises MUC16's protective barrier to facilitate infection

In non-small cell lung cancer, high expression of MUC16 is associated with family history of familial lung cancer (FLC) and indoor air pollution [4]. Polluted air contains high levels of carcinogens, which may be linked to mutations in the MUC16 gene. Some mutations in the MUC16 gene induce overexpression of MUC16, both at mRNA and protein levels. Overexpression of MUC16 can promote the growth, migration, invasion, and cisplatin resistance of lung cancer cell [3]. MUC16 up-regulates the expression of Testis-Specific Y-Encoded-Like Protein 5 (TSPYL5) in lung cancer through the JAK/STAT3/glucocorticoid receptor (GR) signaling pathway, and the upregulation of TSPYL5 inhibits the expression of p53 and related genes, thus promoting the growth of lung cancer cells and cisplatin resistance (Fig. 4) [63].

Endoplasmic reticulum oxidoreductase 1L (ERO1L), a key enzyme in disulfide bond formation, significantly affects MUC16 expression in lung cancer. ERO1L regulates Interleukin 6 receptor (IL-6R) by disulfide bond to promote the expression of muc16 and secretion of CA125. ERO1L directly affects the secretion of IL6R by affecting the formation of IL6 disulfide bonds. IL-6R binds to Interleukin 6 (IL-6) and promotes the expression of MUC16 and the secretion of CA125 by activating the NF-κB signaling pathway. The C-terminus of MUC16 (MUC16-C) can promote the expression of IL-6 and promote the formation of IL6 signaling pathway. ERO1L also indirectly promoted the migration of lung cancer cell lines through MUC16-C. Therefore, it can be concluded that ERO1L can simultaneously promote the secretion of IL6, IL6R, and CA125, and form a positive feedback loop with MUC16 to promote the expression of MUC16 and the release of CA125, thereby promoting the migration of lung cancer cells [64].

**Fig. 5:**
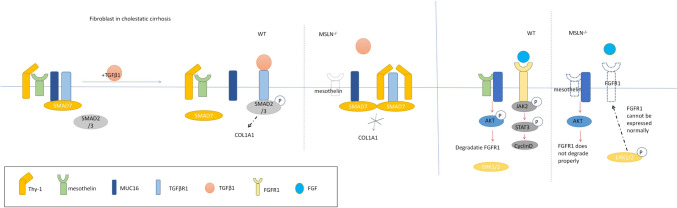
The mechanism of MUC16 in cholestatic hepatic fibrosis. In wild type (WT) aPFs in cholestatic fibrosis, Thy-1 and MUC16 interact with TGFβRI, MUC16, and Thy-1 bind to MSLN (but not to each other), and SMAD7 binds to the complex. TGF-β1 exposure triggers the separation of Thy-1 and MUC16 from TGF-βri, promoting COL1A1 expression. In Msln-/ -APF, the absence of Msln increases the formation of the inhibitory Thy-1-TGFβRI complex and blocks the expression of COL1A1. Fgf-induced pathways differ, with impaired AKT phosphorylation, impaired compensatory ERK1/2 phosphorylation, and altered JAK2/STAT3 activation

MUC16 is overexpressed in the lung tissue of IPF patients and is distributed in pathologically proliferative alveolar type II cells and lung fibroblasts in dimensional foci. Upon stimulation of transforming growth factor-β1 (TGF-β1), MUC16 forms protein complexes with pSmad3, a downstream molecule of the classical TGF-β1 pathway, promoting Smad3 phosphorylation and Smad binding element (SBE) activation. This promotes the transformation of alveolar type II cells into mesenchymal cells and fibroblasts into myofibroblasts, and induces the proliferation of lung fibroblasts, which may contribute to the formation of IPF fibroblast foci [65]. Therefore, MUC16 may be a potential new target for IPF disease (Fig. 5).

### Epidemic keratoconjunctivitis (EKC)

Membrane-associated mucin (MAM) MUC16 regulates the inflammatory response of corneal epithelial cells by restricting the expression of pro-inflammatory cytokines IL-6, IL-8 and TNF-α induced by Toll Like Receptor 2 (TLR2) and Toll Like Receptor 5 (TLR5) [66]. Human adenovirus D (HAdV-D) is often associated with EKC. Adenovirus (HAdV-D37), which causes EKC, but not non-EKC-induced adenovirus (HAdVD19p), has been shown to induce the release of MUC16 (a barrier MAM on the ocular surface) extracellular regions in differentiated human corneal and conjunctiva epithelial cells, which may be one of the means by which HAdV-D37 can infect underlying cells by compromising the MAM glycocalyx barrier of the corneal epithelium. Therefore, blocking the release of MUC16 extracellular region induced by HAdV-D37 may be a novel method to control the transmission of adenovirus keratoconjunctivitis [67].

### Cholestatic liver fibrosis

In biliary obstruction, portal vein fibroblast (PF) proliferates and upregulates the expression of type I collagen, α-smooth muscle actin (α-SMA), and IL-6 to form myoblasts. Type I collagen in early cholestatic liver injury is mainly produced by activated portal fibroblasts (APFs)-derived myoblasts and is an important source of transforming growth factor-β2. We found that the activity of the transforming growth factor βRI-transforming growth factor βRII complex in cholestatic liver fibrosis can be regulated by interaction with the Thy-1-MSLN-MUC16-SMAD7 complex, in which MSLN and MUC16 promote Transforming growth factor-β1 responses in APF, while Thy-1 and Smad7 inhibit transforming growth factor-β1 responses. Deletion of MSLN resulted in overexpression of Thy-1, MUC16, and Smad7, increased formation of the inhibitory Thy-1-TGF-βRI complex, and retained Smad7 at the C-terminus of the MUC16 TGF-βRI receptor. MUC16 is a ligand for MSLN, and MSLN requires MUC16 for intracellular signaling to activate the MSLN/MUC16/AKT pathway, regulating cholestasis-induced proliferation of activated portal fibroblasts/myofibroblasts, but not affecting Fibrotic properties of APFs [68].

## Role of MUC16 in immune processes

MUC16 can help recruit T cells and build a good anti-tumor immune microenvironment, which plays an important role in the immune process.

Since mesothionin polypeptides provided by MUC16 and its ligand MHC were exclusively presented at MHC class II antigens, while human leukocyte antigen (HLA) ligands derived from MUC16 are overexpressed on cancer cells, more than 85% of HLA ligands derived from MUC16 are immunogenic and capable of activating T cells in healthy individuals. Therefore, MUC16 is considered a primary antigen for immunotherapy in EOCs [69].

**Fig. 6:**
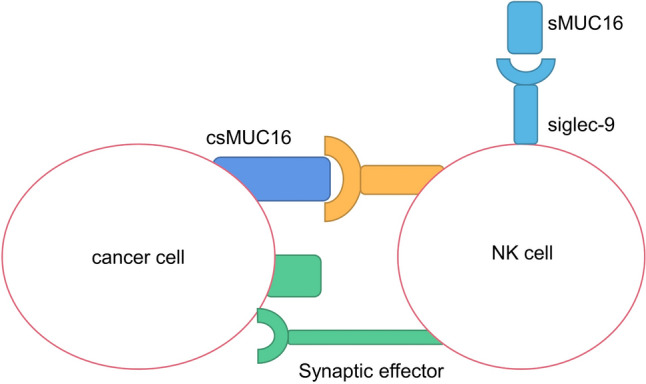
MUC16-mediated inhibition of NK cells in epithelial ovarian cancer. Shed MUC16 (sMUC16) binds to NK cells via Siglec-9, initiating immune suppression prior to NK cell tumor-killing. Cell surface MUC16 (csMUC16) reduces NK cell-cancer cell proximity, diminishing their binding and impairing NK cell-mediated tumor cell killing

The MUC16 binding mucin on the cell surface is called csMUC16, and 50 amino acids in the upper reaches of the transmembrane region are hydrolytically cleaved to obtain the shed mucin sMUC16, both of which can bind to natural killer cell (NK) cells and inhibit NK cells from killing tumor cells. sMUC16 binds specifically to the NK cell subpopulation of epithelial ovarian cancer patients through inhibitory receptor Sigloc-9, causing immune cell suppression to initiate before NK cells kill tumor cells, resulting in impaired anti-tumor immune function. The combination of csMUC16 molecules with NK cells can shorten the distance between NK cells and cancer cells, making them unable to combine, thus reducing the killing of NK cells on tumor cells (Fig. 6) [70, 71].

In endometrial cancer patients with MUC16 mutation, T-cell-mediated immune response was expressed in abundance, T-cell and CD8 + T-cell infiltration increased in the tumor microenvironment, and two interleukin-12 (IL-12)-mediated pathways were up-regulated in T cells and NK cells, suggesting that MUC16 mutation could promote anti-tumor immune response in patients. Many patients with MUC16 mutations carry a large number of passenger mutations, resulting in a higher mutation burden, an enhanced anti-tumor immune response, and a favorable prognosis in the tumor microenvironment. However, MUC16 mutations promote good prognosis and anti-tumor immune response in patients with or without high mutation load [72].

Long-term survivors of pancreatic cancer have a high number of MUC16 neoantigens, much higher than short-term survivors, and MUC16 neoantigens can bind to T cells for specific T cell responses [73]. Patients with somatic MUC16 mutations have a better prognosis and stronger cytotoxic T lymphocyte invasion and antitumor immunity than patients with wild-type MUC16 [72].

## MUC16 has the potential of clinical application as mucin

MUC16 and MUC1, MUC3A, MUC3B, MUC4, MUC12, MUC13, MUC15, and MUC17 belong to the transmembrane mucins, among which MUC1 has been the most extensively and deeply studied. A considerable number of MUC16-related drugs have been developed and applied, which indicates that MUC16 is very important as a clinical therapeutic target. At the same time, due to the similar structure of MUC1 and MUC16 and more studies on the treatment of MUC1, we can also infer that MUC16 has the same huge therapeutic potential. In the following, we summarized the recent progress of MUC16-related treatment and prospected the future development prospect of MUC16.

### Target-specific sites on mucin

Due to the structure of transmembrane mucins and the properties of hydrolytic shedding, drugs that identify mucins in whole or in serum are easy to miss the target, and drug efficacy is relatively low. More precise identification of mucins on tumor surfaces may improve drug efficacy and delivery efficiency.

Targeting the glycosylation sites of mucins is a common means of reducing off-target, such as PankoMab-GEX. PankoMab-GEX (also known as gatipotuzumab) is a humanized IgG1 targeting tumor-associated mucin-1 (TA-MUC1), which is a novel carbohydrate-induced conformational epitope overexpressed only on the surface of tumor cells, and can efficiently recognize tumor cells [74]. PankoMab-GEX has excellent preclinical antitumor activity and has been tested in Phase I and Phase IIb clinical trials (ClinicalTrials.gov Identifier: NCT01222624 and ClinicalTrials.gov Identifier: NCT01899599). Drugs that target MUC16 glycosylation sites also reduce off-target effects, For example, a HIO-refractory, mesothelin-directed ADC (NAV-001) [75] can effectively block the interaction between the intermediate skin protein of tumor cells and CA125 by identifying the glycosylation site of the CA125 binding domain and monoclonal antibody (mAb) AR9.6 and its humanized variant huAR9.6 bind MUC16 to the ErbB (EGF) receptor on the surface of cancer cells by binding to SEA domain 5(a conformational epitope influenced by *O*-glycoylation) on MUC16 to disrupt the binding of MUC16 to the ERbB (EGF) receptor [55, 76]. Both of them can effectively inhibit the growth of tumor cells, but at present only stay in pre-clinical experiments, and the relevant efficacy needs to be further verified in clinical trials.

In addition to the glycosylation site, the C-terminal of mucin is also one of the important recognition sites. Mouse mAb5E6 can improve drug efficacy by more accurately recognizing MUC16-CTER on the surface of tumor cells (the endogenous 114-amino acid carboxy-terminal fragment of MUC16 retained on the cell surface) [77]. A partial human version of mAb5E6 (chimeric mAb5E6/ch5E6) has also been developed, pending further clinical validation.

### Changing the way drugs are delivered

In addition to the traditional reduction of off-target effects, researchers have also developed different drug delivery methods to deliver drugs in smaller volumes and in a more covert manner to improve the effectiveness of drug treatment. The introduction of MUC16 promoter-driven gro-α shRNA vectors into ovarian cancer cells via nanoparticle complexes rich in follicle-stimulating hormone (FSH) peptides is a novel form of drug delivery that can deliver drugs more efficiently and can reduce the secretion of gro-α protein in ovarian cancer cells to inhibit tumor growth [78]. Conditionally replicating tumor adenovirus (CRAd) can package a 5kda DNA fragment upstream of the MUC16 promoter, enabling precise replication and cutting of CA-125 in cancer cells [79]. In addition, the treatment of tumor necrosis factor-associated apoptosis-inducing ligands (TRAIL) is also a new direction, such as Meso-TR3 of human recombinant TRAIL targeting MUC16 (CA125) that can transport TR3 to cancer cells to play a role [80].

### Bispecific antibody

Bispecific antibody refers to antibodies that can recognize two antigens. It is often used in combination with chimeric antigen receptor-T cell immunotherapy (CAR-T) and other immunotherapies to attract and activate immune cells, guide immune cells to find tumor cells and trigger immune response to kill tumor. Mucin is often used as one of the targets of bispecific antibodies.

Conventional bispecific antibodies are often used in combination with CAR T cell therapy. For example, REGN4018 targeting MUC16/CD3 and MUC16/CD28 targeting MUC16 are both bispecific antibodies binding to MUC16 proximal membrane region (MUC16△), which can effectively bind MUC16 and induce T cells to redirect to kill tumor cells. Phase II trials of both are ongoing in advanced ovarian cancer (ClinicalTrials.gov Identifier: NCT04590326) [81].

A modified MUC16-specific CAR T cell (4H11) secreting a bispecific T cell adapter (BiTE) constructed from the WT1-derived epitope RMFPNAPYL (RMF) presented by HLA-A2. This cell can recognize both Muc16 on the surface of tumor cells and Wilms tumor (WT)1 inside cells, and shows stronger anticancer activity against cancer cells with low expression of Muc16 than 4H11 CAR T cells [82]. MUC16^ecto^-BiTE, which also acts as BiTE, specifically recognizes the retention domain of MUC16 (CA-125) and CD3, and is currently mainly used in combination with CAR T cells [83].

In addition to CAR T cells, bispecific antibodies can also be used in combination with other immunotherapies. Since MUC16 can promote tumor progression by directly inhibiting NK cells in tumors, we hypothesized that bispecific antibodies combined with NK cells may be more effective. There is a precedent for the use of MUC1-targeting bispecific antibodies in combination with NK cells. Muc1-bi-1 is a bispecific antibody targeting Muc1 and CD16, and MUC1-BI-2 is its humanized form. Muc1-Bi antibodies recruit NK cells to drive effective and specific cell killing of MUC1-overexpressed tumor cells [84].Therefore, we hypothesized that bifocal antibodies targeting both MUC16 and CD16 would have similar effects in tumors. MUC16 can directly inhibit NK cells in tumors through spatial distance and promote tumor progression (Fig. 6). We hypothesize that specific antibodies targeting MUC16 and CD16 can increase the distance between MUC16 and the MUC16 receptor on NK cells by recognizing the MUC16 antibody on tumor cells and the CD16 antibody on NK cells respectively, so that the NK cells can function normally.

### Improvement of CAR-T therapy

Immunotherapy is an important part of cancer treatment [85]. CAR-T cells are a new therapeutic strategy, but single CAR T therapy is not effective, so the improvement of CAR T therapy is an important direction of future treatment.

One of the important methods to improve the efficacy of CAR-T is to improve the recognition efficiency and reduce the off-target, and increasing the recognition target is the most important method. For example, in EOC mice, PD1-anti-MUC16 CAR T cells showed stronger anti-tumor activity than single CAR T cells [86]. PRGN-3005, which simultaneously expresses CAR, membrane-bound interleukin-15 (mbIL15) and kill switch targeting the unshed part of MUC16, demonstrated its specificity and effectiveness in the treatment of ovarian tumors in vitro. Phase I clinical trials are ongoing (ClinicalTrials.gov identification number: NCT03907527). The core binding fragment of mesolepin can connect with 4-1BB and CD3ζ signal fragments to form CA125-targeted chimeric receptor (CR). The targeted recognition ability and therapeutic efficacy (IL-2 and interferon γ (IFN-γ) release) of CR and CAR group T cells were significantly improved compared with single CAR T cells [87]. In addition to increasing the recognition target, improving the recognition accuracy is also a feasible method to improve the recognition efficiency. Anti-tn-muc1 CAR T cells have anti-tumor effects in pancreatic cancer, suggesting that the recognition of glycosylated mucin phenotypes can not only promote the therapeutic efficacy of conventional antibodies and other drugs, but also improve the therapeutic effect of CAR T. Identifying epitopes on MUC16 may be an important means to improve the efficacy of CAR-T in the future.

Another important way to improve the efficacy of CAR-T is to improve the killing effect of CAR-T cells against tumors. CAR targets MUC16ecto 4H11-28z (4H11-28z), a gene encoding the fusion of human interleukin-12 (IL-12) p35 and p40 subunits (flex-IL-12 (fIL-12)), and truncated EGFR 4H11-28z/fIL-12/EFGRt CAR cells combined with EGFRt can secrete IL-12 as a signal, which synergies with T cell receptor activation signal and CD28 co-stimulatory signal, optimizes the clone expansion and effector function of T cells, and secretes higher levels of IFN-γ and IL-12. They showed a stronger therapeutic effect than conventional CAR T cells [88]. On this basis, we have attempted to develop autologous T cells targeting the combination of MUC16ecto and cyclophosphamide (MSKCC: IL-12), but this phase I clinical trial is currently ongoing (ClinicalTrials.gov ID: NCT02498912).

In addition to 4H11-28z/fIL-12/EFGRt CAR T cells, gene modification of MUC1-targeting CAR T cells to co-express the co-stimulatory receptor (TR2.41BB) also showed some enhancement. Co-stimulatory receptor (TR2.41BB) not only induced apoptosis of myeloid suppressor cells (MDSC) at the tumor site by activating tumor necrosis factor associated apoptosis-inducing ligand receptor 2 (TR2), but also transmitted a second co-stimulatory signal to CAR T cells through 41BB endodomain to better activate T cells. It has excellent anti-tumor potential in breast tumors containing immunosuppressive tumor microenvironment and pro-tumor myeloid-derived suppressor cell (MDSC), promoting tumor microenvironment remodeling and T cell proliferation at the tumor site [89]. This indicates that improving the efficacy of CAR-T cells targeting MUC16 is also an important means to improve the efficacy of CAR-T in the future, and the specific methods need to be further studied in the future.

### CAR-NK cell therapy

At present, the main clinical immune cell-related therapy is CAR-T cells, but the traditional CAR-T cell therapy is not effective. In addition to improving CAR-T cells, researchers have also explored the possibility of applying schemes to other immune cells in recent years. In addition to the effective killing of tumor cells by the combination of MUC1 bispecific antibody and NK cells, the induced pluripotent stem cells expressing MUC1-targeting CAR NK cells (IPSC-derived MUC1-targeting CAR NK cells) also have a significant effect on human oral tongue squamous cell carcinoma (OTSCC) [90]. We speculated that CAR-NK cells targeting MUC16 could also have some effect in cancer therapy. However, considering that the inhibition of MUC16 on NK cells is directly inhibited through spatial distance, it is possible that tumor cells may also be unable to bind to inhibitory receptors after binding with MUC16 receptors of CAR-NK, resulting in the inability of CAR-NK cells to play the role of killing tumors. The specific efficacy of CAR-NK cells targeting MUC16 needs to be explored in more experiments.

### Vaccines

In addition to the direct therapeutic effects of drugs as targets, mucin can also be used to prepare vaccine-assisted therapy.Muc16-related vaccines are primarily anti-human CA-125 recombinant antibodies (Abagovomab (ACA125)), which can induce an immune response against ovarian cancer, but with modest efficacy [91] (ClinicalTrials.gov identifier:NCT00418574). TG4010, which encodes MUC1 and interleukin 2 (IL2), is a poxvirus-based modified vaccine that enhances the efficacy of chemotherapy in advanced NSCLC (ClinicalTrials.gov identifier: NCT00415818) [92]. Therefore, although ACA125 has no therapeutic effect when used alone, it may be used in combination with chemotherapy drugs to increase the therapeutic effect of chemotherapy and become a new adjuvant therapy.

## Discussion and perspectives for the future

MUC16, a pivotal mucin, plays a vital role in safeguarding epithelial cells and is implicated in diverse diseases, particularly cancer. Functioning as a biomarker, it facilitates early cancer detection, subtype identification, and impacts immunotherapy and prognosis.

MUC16 interacts with various elements, impacting tumor progression through different mechanisms and is an important target for potential clinical treatment. In ovarian and pancreatic cancers, it associates with mesenchymal proteins, β-galactoside binding lectin, E- and L-selectin, and FAK, fostering tumor advancement. MUC16 activates the PI3K/AKT pathway, promoting tumor proliferation and invasion. GLUT1, a downstream target, is regulated by MUC16, influencing tumor cell proliferation through glucose metabolism. Mutated KrasG12D regulates MUC16/CA125 via the ERK/FBW7/c-Myc axis. The extracellular part and truncated *O*-glucose chain of MUC16 contribute to tumor progression. MUC16's high CTD expression and circMUC16 exacerbate EOC and PC cell invasion and metastasis.MUC16 is implicated in IPF and cholestatic hepatic fibrosis, potentially through abnormal fibroblast formation and IL-6 secretion. Mutations in MUC16, seen in gastric and endometrial cancers, correlate with higher TMB and improved prognosis.

MUC16 is associated with immunity, and sMUC16 and csMUC16 promote tumor progression by inhibiting NK cells. Mutated MUC16 helps to enhance T cell infiltration. Targeting MUC16 holds promise in cancer treatment, although complete signaling pathways and microenvironment effects warrant further exploration. MUC16 remains a critical player in disease progression, offering potential therapeutic avenues.

## Data Availability

Not applicable.

## Abbreviations

MUC16: Mucin 16
SEA: Sperm, enterokinase, and agrin
CA125: Carbohydrate antigen 125
NLS: Nuclear localization signal
ERM: Ezrin/radixin/moesin
DC-SIGN: (dendritic cell-specific ICAM-grabbing non-integrin)
IPF: Idiopathic pulmonary fibrosis
ILD: Interstitial lung disease
RA-ILD: Rheumatoid arthritis-related interstitial lung disease
CUP: Cancer of unknown primary
EBV: Epstein–Barr virus
EBV-LELCC: Epstein–Barr virus associated lymphoepithelioma-like cholangiocarcinoma
IHCC: Intrahepatic cholangiocarcinoma
PMME: Primary malignant melanoma of the esophagus
PMP: Pseudomyxoma peritonei
OSCA: Ovarian serous cystadenocarcinoma
SNPs: Single nucleotide polymorphisms
EOC: Epithelial ovarian cancer
IRVF: Intrarenal venous flow
AHF: Acute heart failure
NT-proBNP: N-terminal pro-brain natriuretic peptide
TR: Tricususal regurgitation
HF: Heart failure
SV: Slice variants
BUN: Blood urea nitrogen
PDAC: Ductal carcinoma of the pancreas
EC: Endometrial cancer
HE4: Human Epididymis 4
LVSI: Lymphatic space infiltration
LNM: Lymph node metastasis
TMB: Tumor mutation burden
TML: Tumor mutation load
PD-1: Programmed death 1
MUC4: Mucin4
TTN: Titin
OS: Overall survival
TIM: Tumor immune microenvironment
PD-L1: Programmed cell death 1 ligand 1
NSCLC: Non-small cell lung cancer
ICI: Immune checkpoint inhibitor
HCLE: Human limbal epithelium
HIV: Human immunodeficiency virus
MSLN: Mesothelin
DKK1: Dickkopf-related protein 1
SGK3: Serum and glucocorticoid-regulated kinase 3
FOXO3: A forkhead box O3
Bcl-2: B-cell lymphoma 2
GLUT1: Glucose transporter 1
AKT: AKT Serine/Threonine Kinase 1
PI3K: Phosphatidylinositol 3'-kinase
P120ctn: P120-catenin
CTD: Cytoplasmic tail
Rho: Ras homolog
CircMUC16: Circular MUC16
ATG13: Autophagy Related 13
RUNX1: RUNX Family Transcription Factor 1
MMP7: Matrix metalloproteinase-7
MAPK: Mitogen-activated protein kinase
EMT: Epithelial mesenchymal transformation
FBW7: F-box and WD repeat dominium containing 7
miR-29a: MicroRNA-29a
C1GALT1: Core 1 Synthase, Glycoprotein-N-Acetylgalactosamine 3-Beta-Galactosyltransferase 1
GSK3b: Glycogen Synthase Kinase 3 Beta
TF/T: Thomsen–Friedenreich
Tn: Thomsen–nouvelle
FAK: Focal adhesion kinase
FERM: Four-point-one, ezrin, radixin, moesin
ILK: Integrin-linked kinase
JAK2: Janus Kinase 2
LMO2: LIM domain Only 2
NANOG: Nanog Homeobox
NRP2: Neuropilin 2
FLC: Familial lung cancer
TSPYL5: Testis-Specific Y-Encoded-Like Protein 5
GR: Glucocorticoid receptor
ERO1L: Endoplasmic reticulum oxidoreductase 1L
IL-6R: Interleukin 6 receptor
IL-6: Interleukin 6
MUC16-C: C-terminus of MUC16
TGF-β1: Transforming growth factor-β1
SBE: Smad binding element
PF: Portal vein fibroblast
α-SMA: α-Smooth muscle actin
APFs: Activated portal fibroblasts
EKC: Epidemic keratoconjunctivitis
MAM: Membrane-associated mucin
TLR: Toll Like Receptor
HAdV-D: Human adenovirus D
HLA: Human leukocyte antigen
NK: Natural killer
IL-12: Interleukin-12
CRAd: Conditionally replicative oncolytic adenovirus
TRAIL: Tumor necrosis factor-associated apoptosis-inducing ligand
mAb: Monoclonal antibody
PBMC: Peripheral blood mononuclear cells
PNP: Plasma nanoparticles
MDSC: Myeloid-derived suppressor cells
